# Progressive Neurodegeneration From Anoxic Injury Following Perioperative Cardiac Arrest

**DOI:** 10.7759/cureus.115034

**Published:** 2026-08-23

**Authors:** Hemant K Pandey, Kinsey J Gudenkauf, Visweshwar Swaminathan

**Affiliations:** 1 Neurology, Brain and Spine Center, Chandler, USA; 2 Neurology, A.T. Still University's Kirksville College of Osteopathic Medicine (ATSU-KCOM), Mesa, USA; 3 Medical School, Arizona State University, Tempe, USA

**Keywords:** anoxic injury, bile duct surgery, cardiac arrest, delayed post-hypoxic leukoencephalopathy, neurodegeneration

## Abstract

Severe hypoxic-ischemic brain injury following cardiac arrest can result in persistent and evolving neurologic, cognitive, and visual impairment, with later clinical decline that may be difficult to distinguish from superimposed neurodegenerative disease. A prolonged and evolving pattern of neurologic, cognitive, and visual impairment is documented in a woman throughout her 70s and 80s who survived a perioperative cardiac arrest requiring an extended resuscitation interval. Her recovery was complicated by Anton syndrome, prosopagnosia, gait apraxia, chronic microvascular disease with progressive atrophy, intermittent episodes of delirium during superimposed infections, and later neuroimaging findings suggestive of Alzheimer-type degeneration. This case illustrates the dynamic trajectory of severe anoxic encephalopathy, the challenge of differentiating delayed post-hypoxic injury from superimposed neurodegenerative disease, and the need for sustained multidisciplinary care extending well beyond the initial insult. Serial cognitive assessments over nearly a decade further demonstrate the patient’s initial severe impairment, subsequent partial cognitive recovery, and later persistent moderate impairment, while the evolution of the neuroimaging raises the important question of whether the changes found represented chronic post-anoxic injury, Alzheimer's disease, or a combination of both.

## Introduction

Post-hypoxic brain injury occurs following prolonged cardiac arrest when sustained cessation of cerebral blood flow causes global oxygen deprivation. This results in diffuse neuronal injury, selective vulnerability of metabolically active brain regions, and, in severe cases, permanent neurologic deficits. While early mortality and short-term cognitive outcomes after cardiac arrest have been extensively investigated, far less is known about survivors followed for a decade or longer. Delayed post-hypoxic leukoencephalopathy (DPHL), a progressive white matter degeneration occurring weeks to months after recovery, remains underrecognized and may evolve for years as oligodendrocytes exhibit delayed apoptosis and impaired remyelination [1]. Because DPHL is typically diagnosed based on a characteristic delayed clinical and radiologic evolution, a definitive diagnosis cannot be established in this case given the lack of available early post-arrest MRI for comparison. In addition, aging survivors of global anoxia appear to have heightened vulnerability to later-life neurodegenerative processes, including Alzheimer's disease pathology [2].

A woman who survived a profound postoperative anoxic insult requiring extensive resuscitation was followed clinically for more than 10 years. Her course demonstrated early severe deficits, partial recovery, a period of stabilization, and later decline associated with progressive white matter disease and Alzheimer-type atrophy on volumetric MRI, underscoring the overlapping influences of post-hypoxic injury, microvascular disease, recurrent delirium, and age-related neurodegeneration. Serial cognitive testing and prolonged multidisciplinary rehabilitation provide additional context for understanding this trajectory and the distinction between persistent sequelae of the original hypoxic injury and possible later neurodegenerative disease.

## Case presentation

A previously independent woman, age 74, underwent an endoscopic retrograde cholangiopancreatography (ERCP) in 2013 that was later complicated by massive intra-abdominal hemorrhage. Approximately 10 hours after the procedure, she suffered a cardiac arrest. Return of spontaneous circulation occurred after approximately 11 minutes of resuscitative efforts, after which she was taken back to the operating room. A laceration to the mesenteric vein was identified and clipped. She was then immediately admitted to the intensive care unit and required mechanical ventilation and prolonged critical care. Following her intensive care hospitalization, she was discharged to a long-term care facility for approximately two months. She subsequently underwent approximately one month of inpatient acute rehabilitation followed by approximately two months in a skilled nursing facility before returning home with her husband. During this early recovery period, she received physical therapy, occupational therapy, speech and language therapy, and visual therapy.

Approximately four months after the cardiac arrest, her earliest documented standardized cognitive assessment showed a Montreal Cognitive Assessment (MoCA) score of 10/30, consistent with substantial global cognitive impairment. During this early post-arrest period, she demonstrated profound gait instability and bradykinesia, impaired reading and line-tracking ability, prosopagnosia, and constructional apraxia. She also exhibited symptoms consistent with Anton syndrome, including denial of visual deficits despite markedly impaired functional vision. Visual hallucinations were present during the early days of recovery, likely reflecting occipital cortical dysfunction typical of post-hypoxic injury [3]. The brain MRI obtained approximately four months after the cardiac arrest could not be retrieved because the imaging center where it was performed has permanently closed. This represents an important limitation of the case, as the absence of the initial MRI precludes direct comparison of the initial hypoxic-ischemic abnormalities with the patient’s subsequent neuroimaging findings. Accordingly, the characteristic radiologic evolution that would help establish cannot be assessed in this patient. Although progressive white matter injury was considered as a potential mechanism for the later white matter abnormalities, the available imaging and clinical record do not establish DPHL as a definitive component of her course.

After returning home during the first year following the cardiac arrest, she continued physical therapy, speech therapy, cognitive therapy, and ocular training and vision therapy with an optometrist. Speech therapy was later discontinued because of limited progress, while physical, cognitive, and visual rehabilitation continued as clinically indicated. Approximately one year after the cardiac arrest, prism lenses were prescribed to assist with her visual dysfunction.

Pharmacologic treatment for cognitive impairment was also initiated during the early post-arrest period. Memantine 10 mg twice daily was started, followed closely by donepezil 10 mg daily. She initially tolerated both medications well. Donepezil was subsequently discontinued approximately nine years later, while memantine was continued as part of her cognitive management.

Approximately one and a half years after the cardiac arrest, formal neuropsychological testing further characterized her cognitive deficits. Testing with the Wechsler Adult Intelligence Scale (WAIS) demonstrated a Full Scale IQ of 69, at the second percentile and indicative of significant difficulty with reasoning, problem-solving, and processing information compared to peers her age. Her Perceptual Reasoning Index was 65, at the first percentile, indicating significant difficulty with nonverbal, visual-spatial, and fluid reasoning tasks. These findings were concordant with her clinically prominent visual, constructional, and visuospatial deficits.

**Figure 1:**
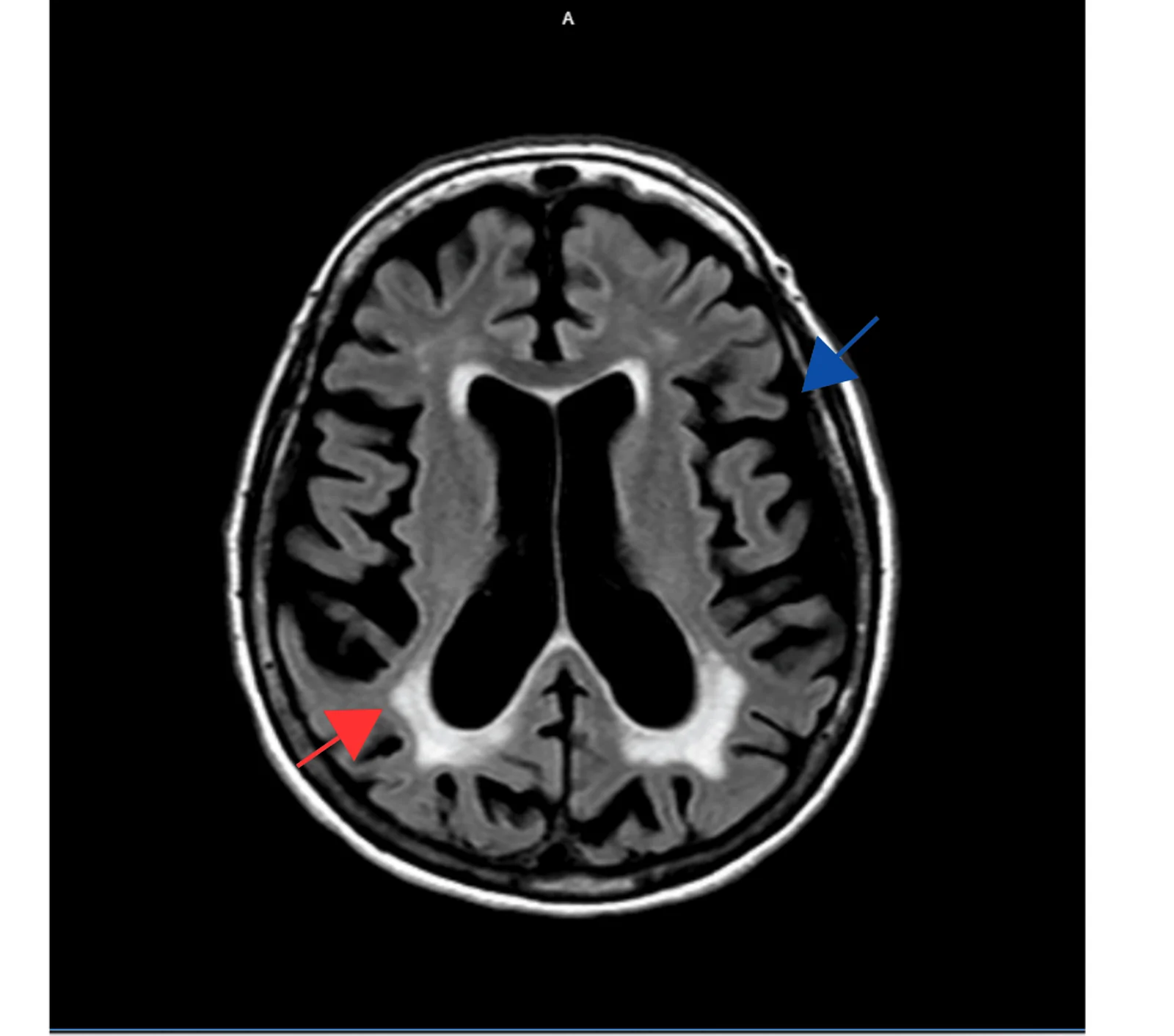
MRI brain: axial T2-FLAIR (fluid-attenuated inversion recovery) Axial MRI demonstrates global cerebral volume loss (blue arrow) and nonspecific periventricular and subcortical white matter hyperintensities (red arrow), consistent with chronic white matter changes.

**Figure 2:**
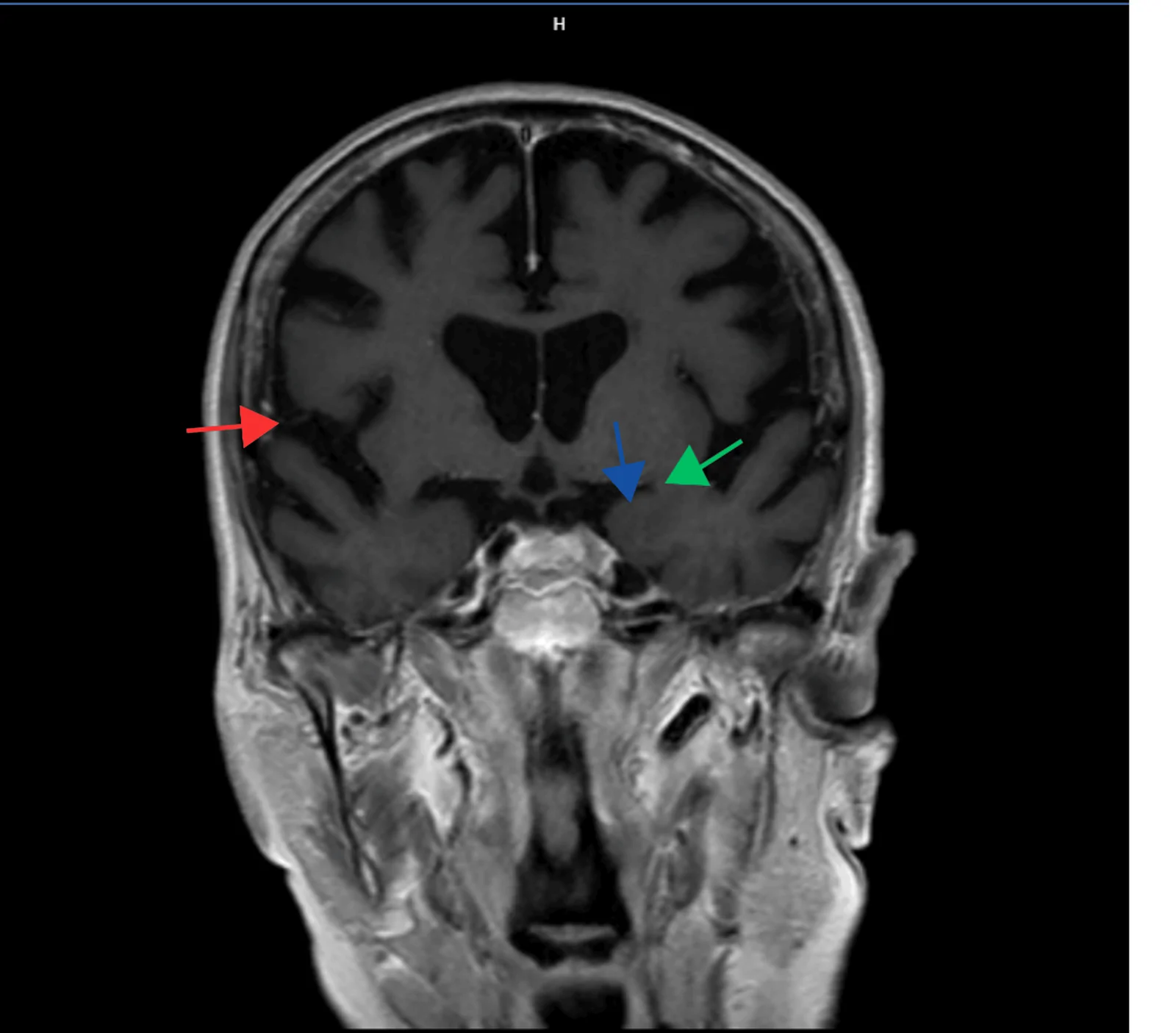
MRI brain: coronal T1 The MRI demonstrates generalized brain-volume loss (red arrow), with demonstration of the hippocampal region (blue arrow) and temporal horn (green arrow).

Neuroimaging obtained during this same period provided the earliest available post-arrest MRI for longitudinal comparison. An MRI brain obtained approximately one and a half years after the cardiac arrest demonstrated global cerebral atrophy and nonspecific periventricular and subcortical white matter changes (Figure 1). Small punctate blooming artifacts were also noted at the bilateral gray-white matter junctions on MRI and were considered compatible with cerebral microhemorrhages in a distribution that may be suggestive of cerebral amyloid angiopathy (CAA). However, these findings were not independently diagnostic of CAA. A dedicated GRE/SWI sequence from this examination was not available for inclusion as a separate figure, limiting further characterization of these susceptibility findings. The available MRI nevertheless provided an important intermediate neuroimaging reference point, including assessment of hippocampal volume and temporal horn size, for comparison with subsequent imaging (Figure 2).

During the first few years following the cardiac arrest, her functional recovery was gradual. She regained the ability to ambulate with assistance and achieved partial independence in activities of daily living. Neuro-ophthalmologic evaluations continued to demonstrate persistently reduced visual acuity in the absence of optic nerve pathology, impaired upgaze, convergence insufficiency, and acquired color vision deficits. Later examinations identified a left internuclear ophthalmoplegia, partial Gerstmann features, confabulation during visual processing tasks, and a suspected left homonymous hemianopia. These findings suggested bilateral parieto-occipital injury with dorsal stream dysfunction and impaired visual recognition networks. During this period, she also experienced intermittent episodes of acute cognitive worsening associated with systemic illness. One episode was characterized by abrupt confusion, inattentiveness, and profound weakness. She returned to her baseline after treatment for a urinary tract infection, and the episode was attributed to infection-related delirium. Several years later, she was hospitalized for influenza A sepsis requiring antimicrobials and supportive care. Her cognitive symptoms again worsened during the acute illness and improved after adequate treatment. Repeat MoCA testing at this time showed a score of 18/30, an improvement from her initial score of 10/30 but with persistent cognitive impairment. This intermediate assessment was consistent with her clinically observed partial recovery and relative functional stabilization rather than complete cognitive recovery.

As she progressed into her late 70s, clinicians observed subtle but progressive worsening of memory despite her earlier period of relative stabilization. She remained free of hallucinations or parkinsonian features, but her visuospatial deficits persisted. She continued to experience particular difficulty with facial recognition and complex visuospatial interpretation. Her gait progressively became slower, with mild shuffling and features consistent with gait apraxia. She retained partial independence in daily tasks but increasingly required support during unfamiliar or visually demanding activities. In her early 80s, her cognitive difficulties became more apparent. She demonstrated increasing difficulty with multistep tasks, more frequent word-finding pauses, and repetition of questions. Her spouse reported increasing dependence. Neurologic examination at this time showed stable cranial nerve function, normal coordination, and a cautious shuffling gait. Approximately nine years after the cardiac arrest, later cognitive assessment demonstrated a MoCA-Blind score of 14/22, again showing persistent impairment across executive, memory, and visuospatial domains. Laboratory studies, including metabolic, infectious, and nutritional assessments, remained within normal limits.

**Figure 3:**
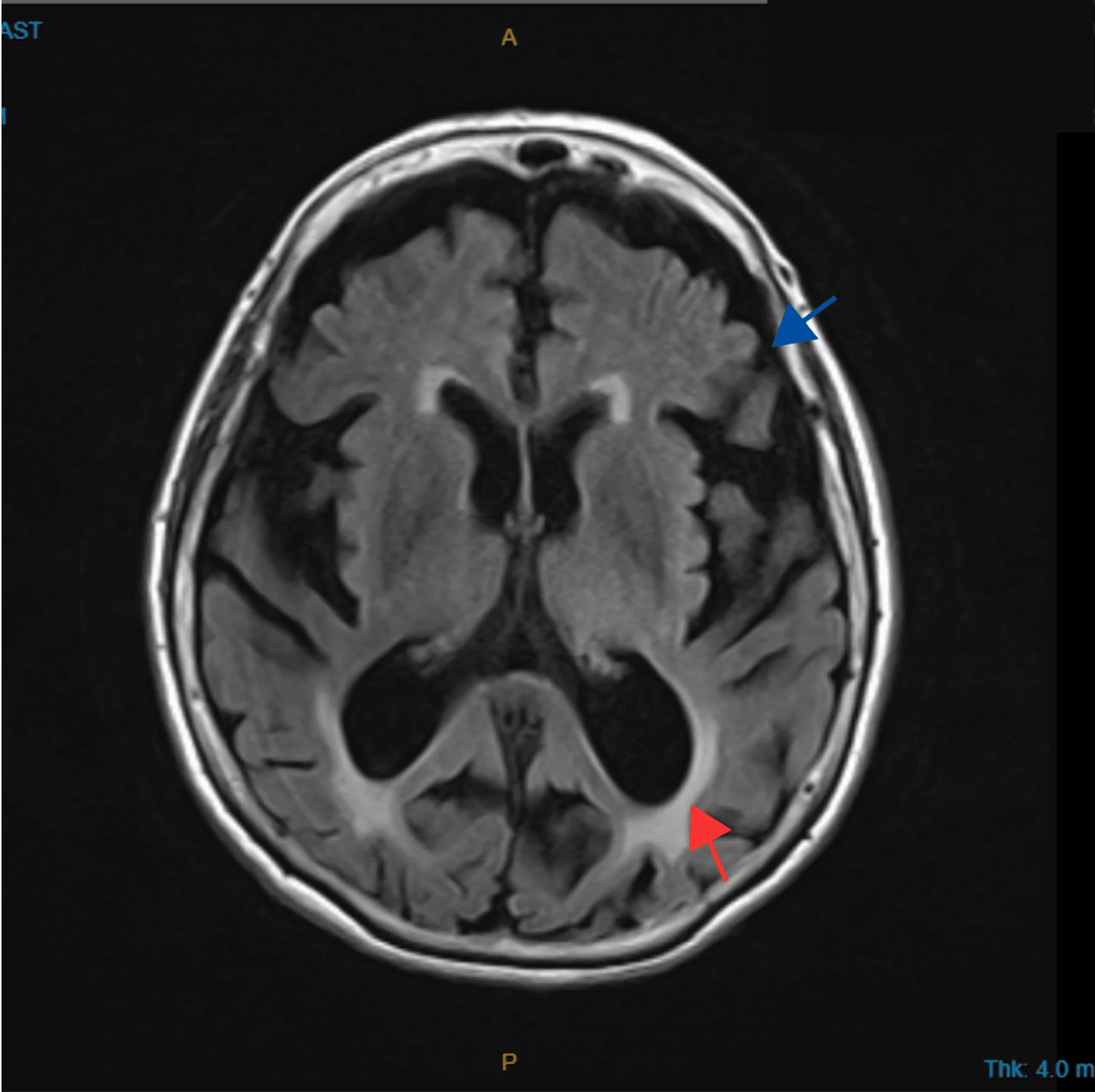
MRI brain: axial T2 T2-FLAIR (fluid-attenuated inversion recovery) Brain MRI demonstrating severe diffuse cerebral volume loss (blue arrow) and moderate-to-severe nonspecific periventricular and subcortical white matter signal abnormalities (red arrow), consistent with chronic microvascular ischemic changes.

**Figure 4:**
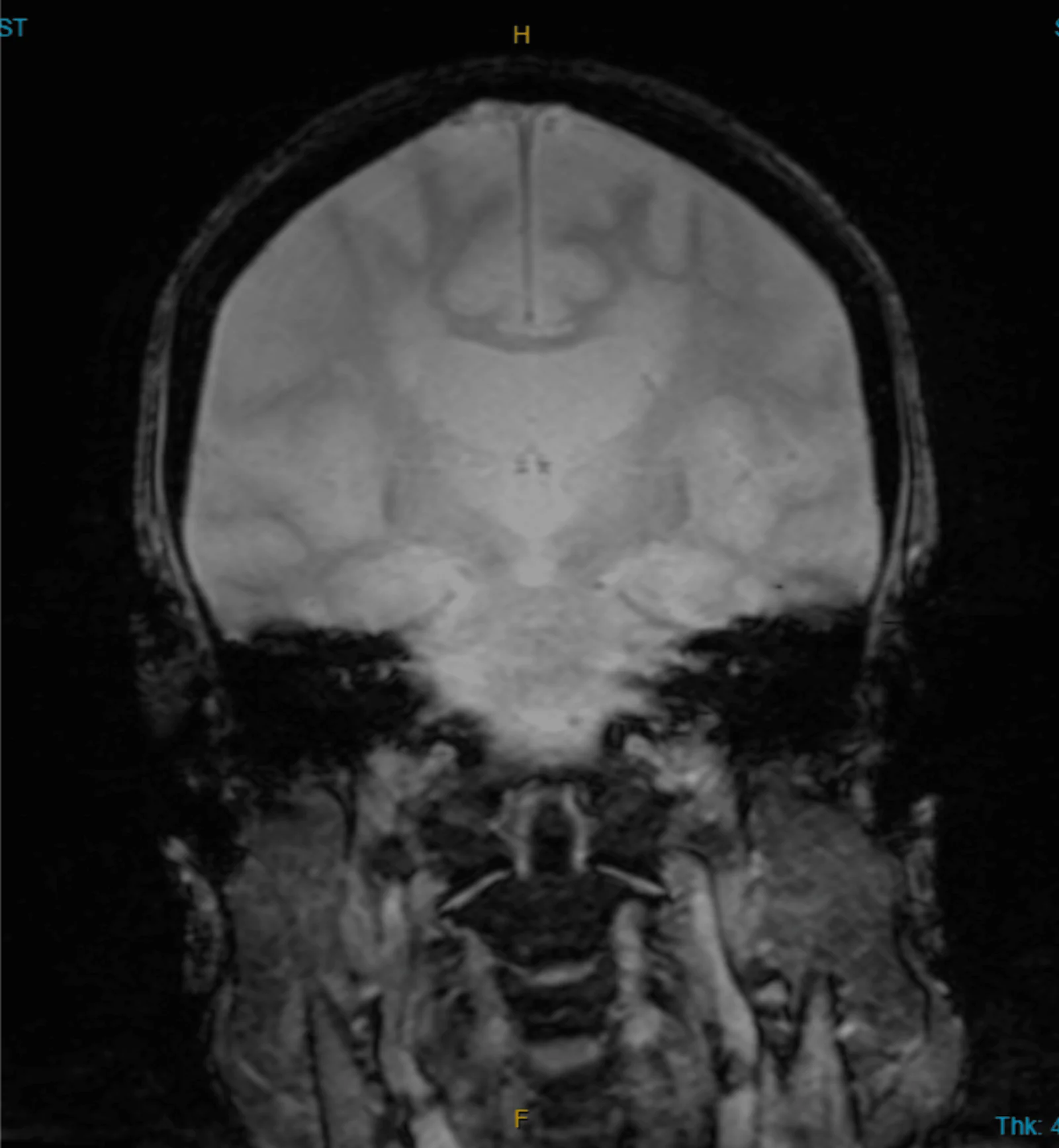
MRI brain: coronal T2 GRE MRI demonstrating susceptibility-sensitive assessment for hemorrhagic abnormalities. No imaging evidence of amyloid-related hemorrhagic co-pathology was identified, including cerebral microhemorrhages or cortical superficial siderosis.

An MRI brain without contrast performed approximately eleven years after the cardiac arrest demonstrated further progression of severe diffuse cerebral volume loss and moderate to severe nonspecific white matter changes consistent with chronic microvascular ischemia (Figure 3). NeuroQuant volumetric analysis additionally demonstrated marked hippocampal volume loss with relative enlargement of the temporal horns. The hippocampal volume was 0.48 cc, corresponding to a corrected normative percentile of 1.00% compared with age-matched controls, while the temporal horn volume corresponded to a normative percentile of 94.00%. A coronal T2 GRE sequence was reviewed for susceptibility-related abnormalities, including hemorrhagic markers associated with the patient's prior anoxic brain injury and the earlier imaging findings that were suggestive of CAA. No imaging evidence of amyloid-related hemorrhagic co-pathology was identified, including cerebral microhemorrhages or cortical superficial siderosis (Figure 4). This finding is relevant to consideration of anti-amyloid therapy should the patient receive a definitive diagnosis of Alzheimer’s disease. Baseline assessment of hemorrhagic markers is important in this setting because of their association with amyloid-related imaging abnormalities and hemorrhagic complications. Although a dedicated 3D T1-weighted sequence was not available for inclusion in the presented MRI images, the NeuroQuant analysis provided quantitative evidence of severe hippocampal volume loss and associated temporal horn enlargement.

The combination of severe hippocampal volume loss and relative temporal horn enlargement raises concern for Alzheimer-type neurodegeneration. However, this interpretation must be considered in the context of the patient’s history of severe global hypoxic-ischemic injury, because the hippocampi and medial temporal structures are also particularly vulnerable to hypoxic-ischemic damage. Thus, the degree of hippocampal atrophy may reflect a combination of chronic post-anoxic injury, age-related neurodegeneration, and possible superimposed Alzheimer's disease [4].

## Discussion

This case demonstrates the prolonged and evolving trajectory of severe anoxic brain injury and highlights the diagnostic challenge of separating chronic post-hypoxic deficits from emerging neurodegenerative pathology. Survivors of global anoxia frequently exhibit early cortical blindness, hallucinations, prosopagnosia, constructional apraxia, and gait impairment due to selective vulnerability of watershed cortical areas and deep white matter tracts [5]. The patient’s parieto-occipital dysfunction, Anton syndrome, prosopagnosia, and visuospatial impairment were consistent with early post-hypoxic cortical injury. DPHL was considered a possible explanation for the later progressive white matter abnormalities because of its recognized delayed clinical and radiologic presentation following hypoxic injury and its association with oligodendrocyte vulnerability and impaired myelin repair [1]. However, the absence of the early post-arrest MRI and the lack of definitive documentation of the characteristic radiologic evolution substantially limit the ability to determine whether DPHL occurred in this patient. Therefore, DPHL should be regarded as a possible explanatory mechanism rather than an established component of her clinical course. Recurrent infections and delirium episodes likely contributed additional periods of cognitive stress and may have further complicated the longitudinal clinical picture.

An additional key feature highlighted here is the emergence of Alzheimer-type neurodegeneration more than a decade after the initial anoxic insult. Global hypoxia may increase long-term risk for Alzheimer pathology by promoting tau phosphorylation, β-amyloid accumulation, and hippocampal susceptibility to delayed neuronal loss [6]. However, the attribution of the patient’s marked hippocampal atrophy specifically to Alzheimer's disease requires caution. The hippocampi and other medial temporal structures are highly vulnerable to hypoxic-ischemic injury, and chronic volume loss in these regions can therefore occur as a consequence of the original cardiac arrest. In this patient, the markedly abnormal NeuroQuant measurements provide objective evidence of severe hippocampal volume loss and associated temporal horn enlargement, findings that are compatible with an Alzheimer-type pattern but are not specific for Alzheimer's disease. Nevertheless, these findings alone cannot definitively distinguish Alzheimer's disease from chronic post-anoxic injury, particularly given the patient’s history of profound global hypoxic-ischemic injury.

The longitudinal evolution of the MRI findings over approximately 10 years provides important context for understanding the patient’s progressive disease course. The earlier MRI, obtained approximately one and a half years following the cardiac arrest, already demonstrated global cerebral atrophy and nonspecific periventricular and subcortical white matter abnormalities. Small punctate blooming artifacts at the bilateral gray-white matter junction were also noted and may be compatible with a CAA-pattern distribution of cerebral microhemorrhages; however, the available findings do not independently establish CAA. The later MRI demonstrated severe diffuse cerebral volume loss with moderate to severe white matter changes consistent with chronic microvascular ischemia. The progression from global atrophy to severe diffuse cerebral volume loss over the long-term interval, together with the marked hippocampal volume reduction on NeuroQuant analysis, supports an evolving multifactorial process, with the later imaging providing additional evidence for a possible superimposed Alzheimer-type process.

The later susceptibility-sensitive imaging provides additional information relevant to the interpretation of the earlier punctate blooming artifacts. On the coronal T2 GRE sequence, there was no imaging evidence of cerebral microhemorrhages or cortical superficial siderosis. Thus, although the earlier distribution of punctate susceptibility artifacts was potentially compatible with CAA, the available imaging does not independently establish a diagnosis of CAA or demonstrate amyloid-related hemorrhagic co-pathology. This distinction is clinically relevant when considering anti-amyloid therapy, for which assessment of baseline hemorrhagic markers is important because of their association with amyloid-related imaging abnormalities and hemorrhagic complications.

The temporal evolution of the neuroimaging findings is therefore particularly important. Imaging obtained immediately after the cardiac arrest, if available, would help establish whether hippocampal, cortical, or watershed abnormalities were already present during the acute hypoxic-ischemic period. The inability to retrieve the early post-arrest MRI remains an important limitation because it prevents direct assessment of the initial distribution and subsequent evolution of the hypoxic-ischemic injury. The overall clinical trajectory of initial severe impairment, partial recovery, prolonged stability, and later decline is consistent with published long-term studies of anoxic brain injury survivors [7]. The diagnosis of superimposed Alzheimer’s disease should be regarded as probable or suggestive rather than established solely on the basis of hippocampal volume. Additional evidence, such as Alzheimer’s disease-specific biomarkers, would strengthen the attribution to a neurodegenerative process.

The serial cognitive assessments provide additional context for this distinction. The initial MoCA score of 10/30 approximately four months after the arrest reflected severe early impairment. Neuropsychological testing approximately one and a half years later demonstrated generally impaired cognitive abilities, with a WAIS Full Scale IQ of 69 and a Perceptual Reasoning Index of 65. The subsequent MoCA score of 18/30 a few years after the event suggests meaningful partial recovery, while the later score of 14/22 approximately nine years after the event demonstrates persistent moderate impairment with some later decline. This pattern of early severe impairment followed by partial recovery and later worsening is compatible with a combination of fixed post-hypoxic injury and possible superimposed age-related or neurodegenerative change, rather than establishing Alzheimer's disease on cognitive testing alone.

This patient presentation underscores the importance of sustained neurologic monitoring, ophthalmologic care, cognitive rehabilitation, and proactive management of infections in long-term survivors. Additionally, it highlights the need for clinicians to remain vigilant for late-onset neurodegenerative disease, which may complicate recovery trajectories and require separate diagnostic and therapeutic approaches. The case supports a multifactorial model of cognitive impairment. The patient’s early visual, parieto-occipital, gait, and executive deficits are strongly temporally linked to the cardiac arrest and are consistent with hypoxic-ischemic injury. Her later white matter changes may reflect chronic post-hypoxic injury, chronic microvascular ischemia, or potentially DPHL, although the available evidence is insufficient to establish DPHL. Her later memory-predominant symptoms and marked hippocampal atrophy raise concern for an additional neurodegenerative process. The NeuroQuant findings provide quantitative support for severe hippocampal volume loss but do not, in isolation, distinguish Alzheimer's disease from chronic post-anoxic injury. Also, because hippocampal injury is a recognized consequence of global hypoxia, it is difficult to distinguish Alzheimer's disease from chronic post-anoxic atrophy. Similarly, the earlier susceptibility findings may have been compatible with CAA but are insufficient to independently establish that diagnosis. This highlights the complexity of interpreting late cognitive decline after severe hypoxic-ischemic injury and the importance of integrating longitudinal clinical, cognitive, and neuroimaging findings when evaluating for superimposed neurodegenerative disease.

## Conclusions

Profound anoxic brain injury can result in lifelong neurologic sequelae that continue to evolve long after the initial insult. This patient’s clinical course illustrates the complex interplay between chronic post-hypoxic cortical and white matter injury, possible delayed post-hypoxic mechanisms, and possible subsequent Alzheimer-type neurodegeneration. As demonstrated in this case, long-term survivors may develop new and progressive neurologic deficits many years after the original injury, underscoring the importance of ongoing surveillance. Comprehensive multidisciplinary care is essential to address cognitive, visual, motor, and behavioral impairments while monitoring for emerging neurodegenerative conditions. Ultimately, severe anoxic brain injury should be viewed not as a static event, but as a dynamic lifelong neurologic disorder requiring continued assessment and adaptive management. In patients with late cognitive decline after cardiac arrest, chronic hypoxic-ischemic injury should always be a consideration. In this case, the markedly reduced hippocampal volume and increased temporal horn volume identified on NeuroQuant analysis provide additional evidence suggestive of Alzheimer-type neurodegeneration, while the patient’s history of severe anoxic injury and chronic microvascular disease underscores the importance of a multifactorial diagnostic framework. Although DPHL remains a possible contributor to the later white matter abnormalities, the absence of the early post-arrest MRI and definitive radiologic evidence of the characteristic evolution prevents this mechanism from being established. Likewise, the distribution of the earlier susceptibility findings may have been suggestive of CAA, but the available imaging does not independently confirm the diagnosis. Recognizing the potential coexistence of post-hypoxic and neurodegenerative pathology may ultimately allow for more individualized long-term care and better support for patients and their families as functional needs evolve over time.
